# Application of Raman Spectroscopy for Sorption Analysis of Functionalized Porous Materials

**DOI:** 10.1002/advs.202105477

**Published:** 2022-01-24

**Authors:** Gregor Lipinski, Kwanghee Jeong, Katharina Moritz, Marcus Petermann, Eric F. May, Paul L. Stanwix, Markus Richter

**Affiliations:** ^1^ Applied Thermodynamics Technische Universität Chemnitz Reichenhainer Straße 70 09126 Chemnitz Germany; ^2^ Fluid Science and Resources School of Engineering The University of Western Australia 35 Stirling Highway Crawley Western Australia 6009 Australia; ^3^ Particle Technology Ruhr‐Universität Bochum Universitätsstraße 150 44780 Bochum Germany

**Keywords:** adsorption, functionalized porous materials, gas separation, Raman spectroscopy

## Abstract

Functionalized porous materials could play a key role in improving the efficiency of gas separation processes as required by applications such as carbon capture and storage (CCS) and across the hydrogen value chain. Due to the large number of different functionalizations, new experimental approaches are needed to determine if an adsorbent is suitable for a specific separation task. Here, it is shown for the first time that Raman spectroscopy is an efficient tool to characterize the adsorption capacity and selectivity of translucent functionalized porous materials at high pressures, whereby translucence is the precondition to study mass transport inside of a material. As a proof of function, the performance of three silica ionogels to separate an equimolar (hydrogen + carbon dioxide) gas mixture is determined by both accurate gravimetric sorption measurements and Raman spectroscopy, with the observed consistency establishing the latter as a novel measurement technique for the determination of adsorption capacity. These results encourage the use of the spectroscopic approach as a rapid screening method for translucent porous materials, particularly since only very small amounts of sample are required.

## Graphical abstract

For the first time, Raman spectroscopy is applied to characterize the adsorption capacity and selectivity of translucent functionalized porous materials. Results for the adsorption of an equimolar (hydrogen + carbon dioxide) gas mixture on three silica ionogels are presented. Validation is carried out by comparison with accurate gravimetric adsorption measurements.

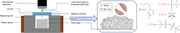

## 1 Introduction

Ionic liquids (ILs) have generated much interest and are widely regarded as suitable materials for various applications due to their unique properties, which include a negligible vapor pressure, high ionic conductivity, chemical and thermal stability and nonflammability.^[^ ^1^, ^2^, ^3^, ^4^ ^]^ The characteristics of these organic salts with low melting points can be attributed to their molecular structure. They consist of low‐molar‐mass anions and cations, and their properties can be tailored by an appropriate selection of the respective ions.^[^ ^5^ ^]^ However, many applications, such as Li‐ion batteries, fuel cells, sensors, and separation processes, would benefit from an immobilization of the IL in a three‐dimensional porous matrix and a vastly increased surface area of the synthesized material.^[^ ^6^, ^7^, ^8^, ^9^ ^]^ Ionogels (IGs) are a group of materials based on silica matrices incorporating ILs with a broad range of applicability.^[^ ^10^ ^]^ In addition to their ability to separate metal ions or their use in chromatography, ILs are known for their ability to dissolve carbon dioxide.^[^ ^11^, ^12^ ^]^ This is utilized in IG polymer membranes that show increased selectivity compared to conventional membranes.^[^ ^13^ ^]^ They can be used for carbon capture and storage (CCS) technologies, since fossil fuels will continue to be relevant to energy conversion for the foreseeable future, and also for the enrichment of fuels for combustion processes obtained from reactions that are used on an industrial scale, for example water‐gas shift reaction.^[^ ^14^ ^]^

Inspired by the promising separation efficiency and selectivity of tailor‐made IGs, along with the large number of possible IG compositions, a fast and reliable method for screening adsorption performance is required to assess novel materials, particularly at times with small sample quantities. Although Raman spectroscopy has been successfully used for the investigation of sorption effects on porous materials and concentration measurements in fluid mixtures, it has not been utilized for the determination of adsorption capacities and the characterization of gas separation processes based on ionogels.^[^ ^15^, ^16^, ^17^, ^18^, ^19^, ^20^ ^]^ Such gels are translucent, which is a decisive precondition to study mass transport inside of a material utilizing light scattering, thus, enabling to derive adsorption capacity that is not easily possible when only studying adsorption at a materials “outer” surface. Here, we demonstrate for the first time that Raman spectroscopy has the potential to be a powerful tool for the rapid determination of the adsorption capacity and selectivity of translucent adsorbents needing only very small amounts of sample (few milligrams). To this end, Raman microscopy was applied to study the adsorption in mesoporous silica ionogels synthesized with imidazolium‐based ionic liquids. Exemplary, an equimolar (hydrogen + carbon dioxide) gas mixture was used to investigate the separation performance of three ionogels. Gravimetric sorption measurements utilizing a magnetic‐suspension balance were conducted independently to verify the spectroscopic results. Since the adsorption capacities determined from the Raman measurements are in good agreement with the results from the reference‐type gravimetric measurements, the method presented here offers a new, more efficient way of quantifying the sorption properties of IGs.

## 2 Results and Discussion

**Figure 1:**
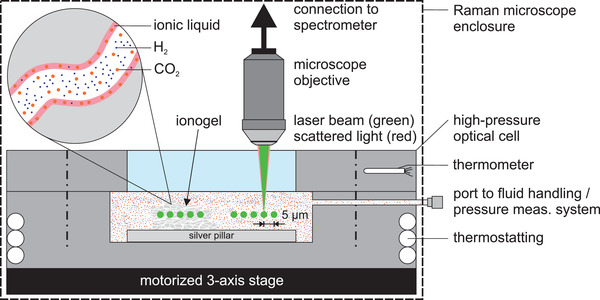
Schematic of the experimental setup for adsorption measurements of an equimolar gas mixture on an IG utilizing a confocal Raman microscope. Spectra are recorded along a single axis at equidistant intervals of 5 µm in the bulk phase (right in the optical cell) and inside the IG particle (left in the optical cell). [We note that this interval was chosen to ensure that there is no overlap between the measured points. As the measurements take place well inside the particle, overlapping points might not be an issue so other intervals would be possible without affecting the result.] Measurements are conducted at *z* = 200 µm, which corresponds to approximately half the height of the investigated particles. The structure of a mesopore is highlighted in the inset. Please note: The details in the schematic are not true to scale.

**Figure 2:**
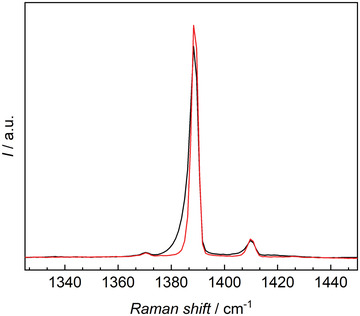
Partial Raman spectra used for the analysis of the adsorption of carbon dioxide, measured for the vapor phase (red) and in IG1 (black) at *T* = 293.15 K and *p* = 50 bar. The main peak can be de‐convolved into free and adsorbed gas peaks as shown in Figure 6.

Details of all experimental procedures and the investigated adsorbents are given in the Experimental section at the end of this paper. Briefly, three silica IGs synthesized with imidazolium‐based ILs were investigated utilizing Raman spectroscopy to determine their adsorption capacity as a function of pressure. For this novel characterization method, line measurements in the IG and the bulk phase of a high‐pressure optical cell were conducted with a confocal Raman microscope and a custom‐made cell (**Figure** **1**). The density of the fluids in the adsorbent was then compared to their density in the vapor phase. For this purpose, the peak area of substance‐specific peaks was determined. The Raman spectral range of (1375–1400) cm^–1^ (**Figure** **2**) was considered for carbon dioxide, while hydrogen peaks were investigated in the range of (4152–4168) cm^–1^. Gravimetric adsorption measurements utilizing a magnetic‐suspension balance were conducted to validate the results. All measurements were carried out at *T* = 293.15 K with an equimolar (hydrogen + carbon dioxide) gas mixture and the pure constituents. (Please note that we do not report results of measurements with hydrogen here as the experimental uncertainty is quite large due to the small signal‐to‐noise‐ratio, thus, the results are not sufficiently significant.) The adsorbents were investigated at a (gauge) pressure of *p* = (1, 2, 3, 4, 5, 10, 20, 30, 40, and 50) bar along the 293.15 K isotherm. Every measurement started at *p* = 1 bar and was then extended to higher pressures. Spectroscopic and gravimetric results are both given as net adsorption, so the results of the new measurement approach can be validated.^[^ ^21^, ^22^ ^]^

**Figure 3:**
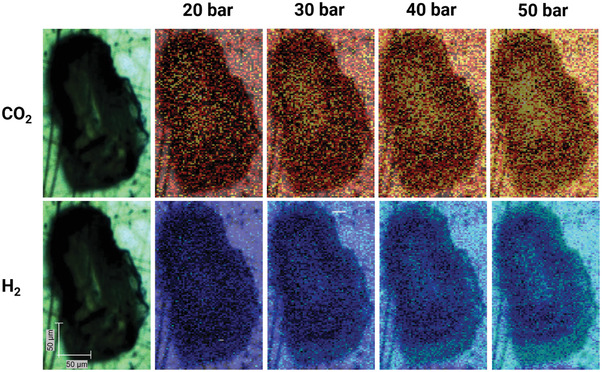
Mapped concentration of carbon dioxide (top) and hydrogen (bottom) on IG1 and photograph of the respective ionogel particle (left). Measurements were conducted at *T* = 293.15 K and *p* = (20, 30, 40, 50) bar. Brighter pixel is assigned to a higher concentration of carbon dioxide and hydrogen, respectively. Each pixel has a size of 2,25 µm^2^.

For the Raman measurements, the samples were first imaged in pure carbon dioxide and pure hydrogen (grid of measurements, scanned via a raster pattern) to provide an overall picture of the potential gas separation capabilities of the investigated ionogel. The results are shown in **Figure** **3**, where a brighter color is assigned to a higher concentration (i.e., Raman peak area) of the respective fluid, and each pixel represents measurement locations separated by 1,5 µm. While an increased concentration of carbon dioxide could be observed in the ionogel, hydrogen showed no apparent adsorption. This is indicated by the higher concentration in the vapor phase surrounding the ionogel while the concentration inside the gel remains almost the same. In contrast, an increased concentration of carbon dioxide is visible in the center of the particle.

**Figure 4:**
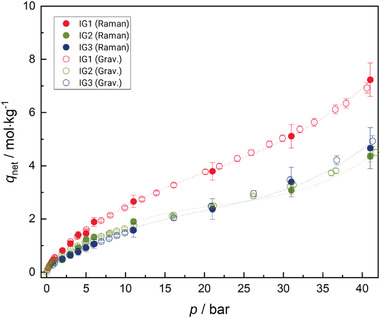
Results of spectroscopic and gravimetric adsorption measurements of pure carbon dioxide on IG1 (red), IG2 (green), and IG3 (blue) along the 293.15 K isotherm. Raman data are shown with filled symbols and gravimetric data with empty symbols. Error bars for the expanded uncertainty of net adsorption *U*_C_(q_net_) with (*k* = 2) are included for every measured (*T*, *p*) state point. Additional lines are plotted to guide the eye.

**Figure 5:**
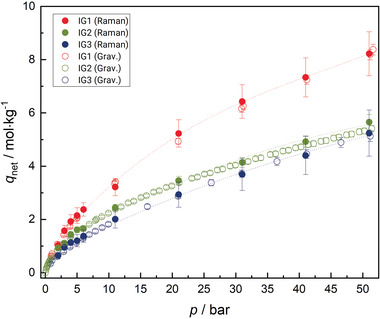
Results of spectroscopic and gravimetric adsorption measurements of an equimolar (hydrogen + carbon dioxide) gas mixture on IG1 (red), IG2 (green), and IG3 (blue) along the 293.15 K isotherm. Results are for carbon dioxide adsorption capacity. Raman data are shown with filled symbols and gravimetric data with empty symbols. Error bars for the expanded combined uncertainty (*k* = 2) of net adsorption *U*_C_(q_net_) are included for every measured (*T*, *p*) state point. Additional lines are plotted to guide the eye.

No adsorption could be determined for hydrogen, so no adsorption isotherm can be shown for this gas species. However, results for pure carbon dioxide and the equimolar gas mixture are shown in **Figures** **4** and **5**, respectively where the net adsorption capacity *q*_net_ (for definitions, see Equations 1 and 7) is plotted versus pressure. (Please note: numerical results are tabulated in the Supporting Information.) Results for pure carbon dioxide show increasing adsorption with increasing pressure. The displayed curves resemble type II isotherms according to the IUPAC classification, which suggests that an overlap of unrestricted monolayer and multilayer adsorption is occurring.^[^ ^23^, ^24^ ^]^ IG1 shows the highest adsorption capacity, whereas the adsorption capacity of IG2 and IG3 is similar. This observation can neither be explained by the specific surface area of the ionogels nor by the solubility of the gas (mixture) in the confined IL. Nevertheless, in the context of ionogels, it is known that the confinement of ILs impacts their gas separation characteristics and can benefit their sorption capacity. Moreover, gas solubility can be impacted by the base material as well as the thickness of the IL layer, however, a straightforward explanation cannot be provided yet; the result rather requires further research. IG3 shows a lower adsorption capacity than IG2 up to a pressure of *p*  =  20 bar but a slightly higher capacity at elevated pressures. Adsorption of the equimolar gas mixture onto the ionogels shows a similar behavior with IG1 having a higher adsorption capacity than the other two ionogels. However, IG2 displays an increased overall adsorption capacity at all investigated state points relative to that measured for pure carbon dioxide. This may be related to the characteristics of the ionogel but the adsorption capacity is generally larger for the mixture. One explanation for this could be that carbon dioxide is a much larger molecule than hydrogen, thus, there are fewer adsorption sites overall occupied when pure carbon dioxide adsorbs. In contrast, if a mixture of carbon dioxide and molecular hydrogen adsorbs, some of the hydrogen molecules could use the space in between the adsorbed carbon dioxide molecules, which are usually highly ordered in the adsorbed phase because of their large quadrupole moment. Hydrogen is entirely nonpolar and could occupy the adsorption sites in between the ordered adsorbed carbon dioxide layers. The shape of the adsorption isotherms in Figure 5 could be assigned to either a type II or type IV isotherm, which is characteristic for mesoporous materials and displays a monolayer adsorption that quickly turns into multilayer adsorption.^[^ ^23^, ^24^ ^]^

The spectroscopic results are in good agreement with the results of gravimetric adsorption experiments for all recorded isotherms. They follow the same trends as the gravimetric reference data and are within the expanded combined uncertainty (*k*  =  2) of both measurement techniques. The measurement uncertainties were estimated according to the Guide to the Expression of Uncertainty in Measurement (GUM) and are indicated in Figures 4 and 5 through error bars.^[^ ^25^ ^]^ The uncertainty of the gravimetric adsorption measurements conducted with a magnetic‐suspension balance was studied before in‐depth, while the uncertainty for the spectroscopic adsorption analysis is reported here for the first time.^[^ ^26^, ^27^ ^]^ Therefore, only the values produced by the gravimetric measurement uncertainty analysis are presented here. An overview of the uncertainty analysis for the Raman measurements is presented in the Experimental section. The estimated uncertainty of the gravimetric measurements is lower than for the Raman adsorption analysis. This is primarily due to the uncertainty of the adsorbent density determination. At high pressures, the larger density of molecules and, therefore, a better signal‐to‐noise ratio reduces the uncertainty of the adsorption capacity measurements using Raman spectroscopy. While the gravimetric results have a relative combined expanded uncertainty in the range *U*_C_(*q*_net_) ≈ (2–4)%, depending on pressure and mass of the sample, the spectroscopic measurements exhibit relative combined expanded uncertainties in the range *U*_C_(*q*_net_) ≈ (4–16)%.

## 3 Conclusion

A new method for the determination of the adsorption capacity of translucent porous materials based on Raman spectroscopy was presented. As proof of function, the adsorption capacity of three silica‐based ionogels was determined in a pressure range from *p*  =  (1–50) bar along the *T*  =  293.15 K isotherm using an equimolar (hydrogen + carbon dioxide) mixture. The uncertainty of the measurements was estimated according to the GUM, and the results were validated by reference‐type gravimetric sorption measurements. The relatively high uncertainty of the spectroscopic measurements is mainly due to the supplementary measurements used to determine the adsorbent density.

This work shows for the first time that Raman spectroscopy can be used as an efficient screening tool for the characterization of porous materials. In contrast to conventional methods, no calibration prior to the measurements is necessary and several fluids can be investigated at the same time when a mixture is used. While other screening methods, such as gravimetric or volumetric approaches, can only determine the total adsorption capacity for a mixture, spectroscopic methods can distinguish between different gas species. It is, therefore, possible to determine if an adsorbent is suitable to separate a gas mixture with a single measurement. Depending on the task, data analysis can be adjusted to give qualitative or quantitative results which, additionally, benefits application‐oriented screening of newly developed materials. This accelerates the characterization process even further, since exact values of the adsorption capacity are only necessary after a successful pre‐scan of the sample.

Although the measurements were successful as a proof‐of‐concept, further improvements to the experimental setup and procedure would help reduce the measurement uncertainty. For example, measurements for each state point should only be conducted after the cell has been flushed with an inert gas to avoid any additional signals from the vapor phase surrounding the adsorbent which was neglected so far. Longer illumination times, the acquisition of more spectra for each evaluation and measurements of temperature and pressure with lower uncertainties would also reduce the overall uncertainty of the determined adsorption capacity. Moreover, to underpin the performance of the novel technique, a larger temperature range and gas mixtures with other species (e.g., natural gas components) have to be studied; we note that for separation of synthetic air (0.79 nitrogen + 0.21 oxygen), our approach worked fine.

## 4 Experimental Section

### Gas Samples and Ionogels

Investigated pure fluids (carbon dioxide, hydrogen, helium) of research‐grade quality were used as received by the supplier, without further purification. The binary mixture for the spectroscopic investigations was also used as received. Its composition was specified by the supplier to be 0.5014 mole fraction hydrogen and 0.4986 mole fraction carbon dioxide with an expanded uncertainty (*k* = 2) in composition of 0.005 mole fraction. However, the binary gas mixture for the nonspectroscopic sorption measurements was prepared gravimetrically using a custom‐made mixture preparation system which has been described before.^[^ ^28^ ^]^ The composition was determined to be 0.5059 hydrogen mole fraction and 0.4941 mole fraction carbon dioxide with an expanded uncertainty (*k* = 2) in composition of 0.0016 mole fraction. Details of all pure fluids are described in **Table** **1**.

**Table 1:** Gas sample information for pure fluids

Chemical Name	Source	Purity	Used for
Carbon dioxide	Coregas	0.99995	Spectroscopy
Hydrogen	Coregas	0.99999	Spectroscopy
Carbon dioxide	Air Products	0.999996	Gravimetry
Hydrogen	Air Liquide	0.99999	Gravimetry
Helium	Air Liquide	0.99999	Gravimetry

For the synthesis of the ionogels, three different 1‐butyl‐3‐methylimidazolium ([Bmim]) based ionic liquids with differing anions were chosen (trifluormethansulfonat [OTf], hexafluorophosphat [PF_6_], bis(trifluormethylsulfonyl)imid [Tf_2_N]) and incorporated into a silica framework based on tetramethyl orthosilicate (TMOS) as a precursor. The investigated ionogels were prepared following the one‐step sol‐gel process and a subsequent supercritical extraction with carbon dioxide in a high‐pressure view cell. High‐purity chemicals were used for the syntheses. Surface area, total pore volume and the average pore diameter of the ionogels were determined through a standard nitrogen adsorption–desorption analysis according to Brunauer, Emmett, and Teller (BET) at *T* = 77 K with a micropore physisorption analyzer (autosorb iQ, Anton Paar, Austria). An overview of the results is given in **Table** **2**. Further characterization and a detailed description of the synthesis can be found elsewhere.^[^ ^29^, ^30^ ^]^

**Table 2:** Results of BET and density measurements for the investigated ionogels. The uncertainty is given as standard deviation

	Ionogel 1	Ionogel 2	Ionogel 3
Ionic liquid	[Bmim][OTf]	[Bmim][PF_6_]	[Bmim][Tf_2_N]
Surface area *A*_sur_ [m^2^ g^−1^]	465.49 ± 3.22	314.74 ± 4.20	481.63 ± 6.88
Total pore volume *V*_tp_ [cm^3^ g^−1^]	1.753 ± 0.007	1.314 ± 0.004	4.515 ± 0.016
Average pore diameter *d*_ap_ [nm]	18.68 ± 0.05	15.34 ± 0.03	33.47 ± 0.18
Skeletal density *ρ* [g cm^−^^3^]	2.22 ± 0.13	2.13 ± 0.04	2.54 ± 0.28

Before measurements, the ionogels were activated inside the respective measurement cell at a temperature of *T*  =  323.15 K in vacuum. A rotary vane pump (RZ 6, Vacuubrand GmbH, Germany) with an ultimate pressure of *p*_ult_ ≤ 2.0 mbar was used prior to the spectroscopic measurements of each gas sample for 1 h. Before the gravimetric measurements, a turbomolecular pump (HiCube 80 Eco, Pfeiffer Vacuum GmbH, Germany) with an ultimate pressure of *p*_ult_ ≤ 0.001 mbar was used for at least 3 h at *T*  =  323.15 K until a stable balance reading when weighing the ionogel could be observed. After the activation process, the mass of the sample was determined with a magnetic‐suspension balance and the sample volume was determined by measuring the buoyancy of the IG in pure helium, so the density could be calculated accordingly. (Note: For the volume determination, it was assumed that helium was not adsorbed onto the ionogel.)

### Raman Adsorption and Selectivity Measurements

All measurements were conducted at *T* = 293.15 K with a binary (0.5014 hydrogen + 0.4986 carbon dioxide) gas mixture and pure fluids, as specified above. Ionogels were placed on a silver pillar inside a custom‐made pressure cell, which was temperature controlled by four Peltier devices and located on a moveable stage under the microscope. Pressure inside the measuring cell was measured with a piezoresistive pressure transducer (PA‐33X, KELLER AG, Switzerland) and temperature measurement was realized with two 100 Ω platinum resistance thermometers (PR‐11‐2‐100‐1/16‐4‐E, OMEGA Engineering Inc., USA). After activation of the IGs, measurements were conducted at a (gauge) pressure of *p* = (1, 2, 3, 4, 5, 10, 20, 30, 40, and 50) bar. Measurements started at *p* = 1 bar and were then extended to higher pressures. At each (*T*, *p*) state point, Raman spectra were acquired with a confocal Raman microscope (InVia Reflex, Renishaw, United Kingdom) with a 532 nm laser excitation source with a maximum power output of 200 mW. The spectrometer had a range of 5400 cm^–1^ and a resolution of ≈1 cm^–1^. Measurements began by focusing the laser at the center of a selected IG particle, ≈200 µm above the surface of the silver pillar to minimize any influence from possible surface interactions. Five points along the x‐axis, each 5 µm apart, were then investigated. The illumination time for a spectrum was 5 s and five accumulations were made for every measurement point. Each measurement was also repeated four additional times to verify the results and lower the uncertainty. Measurements in the vapor phase were conducted at the same height as in the IG (*z* = 200 µm). Acquired spectra were analyzed using the Renishaw WiRE 3.4 software. In addition, scan measurements across a plane were conducted to visualize the selectivity of a selected ionogel. Measurements were performed along a grid with spacing of 10 µm, ≈200 µm above the surface of the silver pillar. One spectrum was recorded for each point of the grid with the same settings as described above. For data analysis, peaks in the range of (1375–1400) cm^–1^ were considered for carbon dioxide, whereas hydrogen peaks were investigated in the range of (4152–4168) cm^–1^.

### Quantitative Raman Adsorption Analysis

As shown before, two measurements were required to calculate the adsorption capacity of the investigated samples. Since net adsorption is reported in this work, data analysis for the calculation of net adsorption is shown below. Please note that this analysis method was explored for translucent materials, but the fundamental approach could be applicable to other forms of adsorption measurements, such as adsorption on 2D surfaces. It can also be extended to report excess or absolute adsorption.

In general, calculations were based on the ratio between the density of adsorbed molecules and the density of the bulk material. The net adsorption capacity *q*_net_ based on the Raman measurements was calculated according to Equation 1, where *ρ*_ads_ is the density of the adsorbate and *ρ*_bulk_ is the density of the adsorbent. In addition, the density from an appropriate equation of state (EOS) is considered

(1) $$q_{\mathrm{net}}=\frac{\rho_{\mathrm{ads}}-\rho_{\mathrm{EOS}}}{\rho_{\mathrm{bulk}}}$$

Within the scope of this work, the EOS of Span and Wagner, Leachman et al. and Kunz and Wagner were used, as implemented in NIST's Reference Fluid Thermodynamic and Transport Properties Database (REFPROP).^[^ ^31^, ^32^, ^33^, ^34^ ^]^ The density from the EOS was recalculated for every state point that was investigated. If the bulk density was not known beforehand, it had to be determined by supplementary measurements.

Calculation of the adsorbed fluid density was done according to Equation 2, where the raw Raman intensity *I*_raw_ from measurements in the adsorbent must be corrected by the experimental correction factor *κ*, and a calibration factor *g*_cal_ is needed, additionally

(2) $$\rho_{\mathrm{ads}}=\frac{I_{\mathrm{raw}}\cdot \kappa }{g_{\mathrm{cal}}}$$

Both values were obtained through analysis of the recorded spectra from the vapor phase measurements and the measurements in the adsorbent, respectively. The calibration factor *g*_cal_ directly relates the measured Raman intensity to molecular density and is specific to the measurement apparatus, since the Raman intensity depends on the characteristics of the spectrometer's excitation and detection systems. It was derived from a linear regression of the recorded vapor‐phase Raman intensities against the calculated density from the EOS for the same (*T*, *p*) state point. The calibration was equal to the slope of the regression. The experimental correction factor *κ* was specific to the adsorbent plus adsorbate system being studied and accounted for differences in optical properties. The calculation of *κ* was not as straightforward, since it required the free and adsorbed gas within the porous material to be resolved through deconvolution analysis of the adsorbent's Raman spectra.

**Figure 6:**
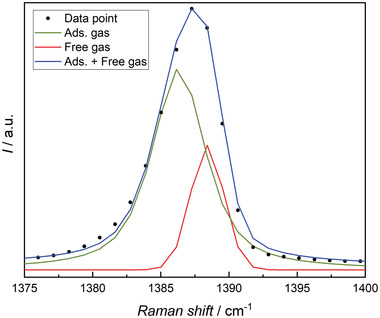
Schematic of a deconvolution analysis. The signal of the Fermi dyad recorded at *T* = 293.15 K and *p* = 50 bar in a range of (1375–1400) cm^−1^ is deconvolved into two separate signals that are assigned to free (red) and adsorbed (green) carbon dioxide molecules. The original Raman data points (black dots) and the sum of the two fitted peaks (blue) are also shown.

Deconvolution analysis could be conducted when a change of the investigated peak in contrast to the vapor phase could be observed, e.g., a peak broadening. It was assumed that the broadening occurs due to an additional sub‐band in the spectrum that can be assigned to adsorbed molecules. Thus, a deconvolution allowed a distinction between free (*I*_raw,free_) and absorbed (*I*_raw,ads_) molecules. An example of the deconvolution analysis of the right peak of the Fermi dyad of carbon dioxide in the range of (1375–1400) cm^–1^ is shown in **Figure** **6**.

For the deconvolution analysis, a linear background was removed and the width and the center (width 2.93 cm^–1^ and 1388.59 cm^–1^, respectively) of the free gas were constrained to match the observed vapor phase peak characteristics. The intensity of the free gas that resides inside of the material was then used to calculate *κ* according to Equation 3

(3) $$\kappa =\frac{I_{\mathrm{vap}}\cdot \gamma_{\mathrm{bulk}}}{I_{\mathrm{raw},\mathrm{free}}}$$

In addition, the porosity of the bulk material *γ*_bulk_ was required, which could be derived from supplementary measurements such as BET physisorption analysis, as used in this work.

### Spectroscopic Uncertainty Analysis

The uncertainty of the Raman measurements was determined according to the Guide to the Expression of Uncertainty in Measurement (GUM). As a first (rather conservative) approach, the combined uncertainty of the adsorbed fluid *U*_C_(*q*_net_) could be estimated according to Equation 4

(4) $$\begin{array}{ccc} U_{C}\left(q_{\mathrm{net}}\right) & = & k\cdot \left[{\left(u_{C}\left(q_{\mathrm{net}}\right)\right)}^{2}+{\left(u_{C}\left(\rho_{\mathrm{ads}}\right)\right)}^{2}+{\left(u_{C}\left(\rho_{\mathrm{EOS}}\right)\right)}^{2}+{\left(u_{C}\left(\rho_{\mathrm{bulk}}\right)\right)}^{2}\right.\\ & & {\left.+\;{\left(u_{C}\left(\gamma_{\mathrm{bulk}}\right)\right)}^{2}+{\left(u_{C}\left(I_{\mathrm{cor}}\right)\right)}^{2}+{\left(u_{C}\left(\kappa \right)\right)}^{2}+{\left(u_{C}\left(g_{\mathrm{cal}}\right)\right)}^{2}\right]}^{0.5} \end{array}$$

The equation consists of several uncertainties that must be calculated beforehand. Beside the uncertainty of the net adsorption *u*_C_(*q*_net_), which is further elaborated in Equation 5, the uncertainty of density of the adsorbed fluid *u*_C_(*ρ*_ads_), the density calculated from a suitable EOS *u*_C_(*ρ*_EOS_), the density of the bulk material *u*_C_(*ρ*_bulk_), the porosity of the bulk material *u*_C_(*γ*_bulk_), the intensity from measurements in the adsorbent *u*_C_(*I*_cor_), the experimental correction factor *u*_C_(*κ*_exp_), and the calibration factor *u*_C_(*g*_cal_) are needed to calculate *U*_C_(*q*_net_)

(5) $$\begin{array}{ccc} u_{C}\left(q_{\mathrm{net}}\right) & = & \left[{\left(\frac{1}{\rho_{\mathrm{bulk}}}\right)}^{2}\cdot u_{C}^{2}\left(\rho_{\mathrm{ads}}\right)+{\left(-\frac{1}{\rho_{\mathrm{bulk}}}\right)}^{2}\cdot u_{C}^{2}\left(\rho_{\mathrm{EOS}}\right)\right.\\ & & {\left.+\;{\left(-\frac{\rho_{\mathrm{ads}}-\rho_{\mathrm{EOS}}}{\rho_{\mathrm{bulk}}^{2}}\right)}^{2}\cdot u_{C}^{2}\left(\rho_{\mathrm{bulk}}\right)\right]}^{0.5} \end{array}$$

The uncertainty of the net adsorption *u*_C_(*q*_net_) was dependent on the uncertainty of the bulk material's density *u*_C_(*ρ*_bulk_), which could lead to a high overall uncertainty of the measurement. The high uncertainty of the supplementary measurements was, therefore, the main source of uncertainty for the Raman measurements. Other sources of uncertainty included the quality of the spectra which explained a higher uncertainty at lower pressures, where a worse signal‐to‐noise‐ratio could be observed.

### Gravimetric Adsorption Measurements

Gravimetric sorption measurements were conducted with a commercially available gravimetric sorption analyzer (IsoSORP, Rubotherm GmbH, Germany, since 2016, TA instruments, USA) with custom improvements to lower the uncertainty of the measurements.^[^ ^26^ ^]^ It incorporated a magnetic‐suspension coupling, which was often utilized for accurate sorption measurements.^[^ ^35^, ^36^, ^37^, ^38^, ^39^ ^]^ The sorption analyzer could be operated over a broad temperature (278.15–423.15 K) and pressure (up to 35 MPa) range. The temperature of the measuring cell was measured with a 100 Ω platinum resistance thermometer (PT‐103, Lake Shore Cryotronics, Inc., USA) calibrated according to the ITS‐90 at the triple point of water (273.160 K), the melting point of gallium (302.915 K), and the freezing point of indium (429.748 K). In addition to the thermometer, a resistance bridge (MKT50, Anton Paar GmbH, Austria) with a calibrated internal resistor (≈400 Ω) was used for the temperature measurement. Pressure measurement was realized with a vibrating quartz‐crystal‐type transmitter (42K‐101, range up to 13.8 MPa, Paroscientific, USA), which was thermostated at *T* = 333.15 K to prevent any condensation of the gas sample. The pressure transmitter was calibrated in situ with a piston gauge (PG‐7601, Fluke Corporation, USA). Weighing of the sample container was conducted with an analytical balance with a readability of 1 µg (WXS206SDU, Mettler Toledo, Switzerland) through the magnetic‐suspension coupling.

Due to the use of a magnetic‐suspension coupling, it was necessary to take the related force‐transmission error (FTE) into account to ensure the best achievable accuracy. Detailed information on systematic investigations regarding the FTE can be found in related work.^[^ ^26^ ^]^ When taking the FTE into account, the expanded combined uncertainty (*k*  =  2) in density measurement was estimated to be 0.020 kg m^−3^. For temperature and pressure measurements (range: from vacuum to 8 MPa), it was estimated to be 16 mK and between (0.1 and 0.7) kPa, respectively.

The apparatus was designed to measure density and adsorption simultaneously. All presented measurements in this study focused primarily on adsorption. Although density measurements were conducted alongside the sorption measurements, it was decided to use the respective EOS for data analysis for pure fluids to provide the highest possible accuracy^[^ ^31^, ^32^, ^33^ ^]^

(6) $$m_{\mathrm{ads}}=\left[\frac{{\left(W_{\mathrm{MP}1}-W_{\mathrm{ZP}}\right)}_{\mathrm{fl}}}{\alpha \cdot \phi_{\mathrm{MP}1}}-m_{\mathrm{bulk}}+\rho_{\mathrm{fl}}\cdot V_{\mathrm{ads}}\right]\cdot {\left(\frac{\rho_{\mathrm{ads}}-\rho_{\mathrm{fl}}}{\rho_{\mathrm{ads}}}\right)}^{-1}$$

Equation 6 was used to determine the adsorbed mass; it consists of expressions for balance readings at magnetic‐suspension positions MP1 (measuring position 1) and ZP (zero or tare position), i.e., (*W*_MP1_ and *W*_ZP_), the balance calibration factor *α*, a correction factor for the FTE *ϕ*_MP1_, mass of the adsorbent and container *m*_bulk_, the densities of the investigated and adsorbed fluid *ρ*_fl_ and *ρ*_ads_ and the volume of the adsorbent and container *V*_ads_. Since the operating principle of the gravimetric sorption analyzer has been described before, it will only be described briefly here.^[^ ^26^, ^27^ ^]^ In total, there were three stable measuring positions, namely zero point (ZP), measuring position 1 (MP1), and measuring position 2 (MP2). At ZP, the balance was tared. Here, only the permanent magnet of the magnetic‐suspension coupling and, thus, the lifting rod were in suspension, neither the sinker nor the sample‐filled container was lifted. When the suspension position was switched to MP1, the permanent magnet got moved upward due to the change of the current in the electromagnet of the magnetic‐suspension coupling. This caused the sample container to be lifted accordingly and being weighed. The permanent magnet was moved even further upward at MP2. In this case, the sample container and the sinker were lifted and weighed which allowed the calculation of the fluid's density. Results from the weighings at ZP and MP1 were needed for the calculation of adsorbed mass according to Equation 6.

The net adsorption capacity *q*_net_ based on the gravimetric measurements was calculated according to Equation 7, where *m*_net_ is the net adsorbed mass, *M*_fluid_ is the molar mass of the investigated fluid, and *m*_P_ is the mass (and *V*_P_ the volume) of the porous material inside the sample container

(7) $$q_{\mathrm{net}}=\frac{m_{\mathrm{net}}}{M_{\mathrm{fluid}}\cdot m_{P}}=\frac{m_{\mathrm{ads}}-\rho_{\mathrm{fl}}\left(V_{\mathrm{ads}}+V_{P}\right)}{M_{\mathrm{fluid}}\cdot m_{P}}$$

Measurements were conducted along the *T* = 293.15 K isotherm, and the pressure inside the measuring cell was regulated by a software‐controlled gas‐dosing system. Balance readings were recorded alongside temperature and pressure every 5 s during the measurements. After activation of the IG sample at *T*  =  323.15 K, the measuring cell was filled with helium to a pressure of *p* = 2 bar and subsequently cooled down to a temperature of *T* = 293.15 K. Then, measurements with helium were conducted at *p* = (70, 75, 80, 85, 90, 95, and 100) bar to determine the volume of the IG sample according to Equation 6. The pressure was increased stepwise, and an equilibration time of ≈30 min was allowed after a change in pressure. Measurements at any given (*T*, *p*) state point lasted 20–40 min. Afterward, the measuring cell was evacuated for 1 h, and the mass of the IG was determined. This measurement was important to minimize the scattering related to Equation 6 since *m*_ads_ was dependent on the balance readings *W*_ZP_ and *W*_MP1_ which had a significant impact on the results due to the low mass of the ionogel samples. The procedure was followed by sorption measurements with other pure gases (hydrogen and carbon dioxide) and the binary gas mixture (0.5059 hydrogen + 0.4941 carbon dioxide). Again, the pressure was increased stepwise by the software‐controlled gas‐dosing system, and an adequate equilibration time of 30 min was allowed after a change in pressure. The measurements were conducted at *p* = (1, 2, 3, 4, 5, 10, 20, 30, 40, and 50) bar to match the spectroscopic investigations and lasted 20–40 min. To investigate possible hysteresis effects, measurements at corresponding (*T*, *p*) state points with pressure decreasing steps were conducted at the same temperature (*T* = 293.15 K). This cycle was also repeated for each IG to test for reproducibility.

## Conflict of Interest

The authors declare no conflict of interest.

## Supporting information

Supporting Information

## Data Availability

The data that support the findings of this study are available in the supplementary material of this article.
