# Supplementary material for: Application of Raman Spectroscopy for Sorption Analysis of Functionalized Porous Materials
Source: Adv Sci (Weinh). 2022 Jan 24;9(9):2105477. doi: 10.1002/advs.202105477 (PMC8948586; doi:10.1002/advs.202105477)
Supplement: Supplementary file 1 — Supporting Information [file ADVS-9-2105477-s001.pdf]

WILEY-VCH

ADVANCED SCIENCE Open Access

Supporting Information

for *Adv. Sci.*, DOI: 10.1002/advs.202105477

Spectroscopic and Gravimetric Measurement Data

Gregor Lipinski, Kwanghee Jeong, Katharina Moritz, Marcus Petermann, Eric F. May, Paul L. Stanwix and Markus Richter**

Supporting Information

Spectroscopic and Gravimetric Measurement Data

Gregor Lipinski, Kwanghee Jeong, Katharina Moritz, Marcus Petermann, Eric F. May, Paul L. Stanwix and Markus Richter**

Table S1 Experimental results for Raman adsorption measurements of pure carbon dioxide on IG1. Recorded temperature, pressure, net adsorption and estimated uncertainties are listed in the table.

T / K	p / bar	q_{net} / mol·kg^{-1}	$U_C(q_{net})$ / mol·kg^{-1}	$U_C(q_{net}) \cdot (q_{net})^{-1}$ / %
293.150	2.0394	0.8129	0.0873	10.742
293.150	3.0385	1.0692	0.0980	9.165
293.150	4.0377	1.3987	0.1345	9.616
293.150	5.0310	1.4554	0.1378	9.471
293.150	6.0342	1.8851	0.1677	8.897
293.150	11.0436	2.6583	0.2332	8.773
293.150	21.0472	3.7927	0.3294	8.686
293.150	31.0419	5.1191	0.4470	8.731
293.150	41.0390	7.2433	0.6309	8.711
293.149	51.0480	10.8567	0.9567	8.812

Table S2 Experimental results for Raman adsorption measurements of pure carbon dioxide on IG2. Recorded temperature, pressure, net adsorption and estimated uncertainties are listed in the table.

T / K	p / bar	q_{net} / mol·kg^{-1}	$U_C(q_{net})$ / mol·kg^{-1}	$U_C(q_{net}) \cdot (q_{net})^{-1}$ / %
293.150	2.0414	0.4552	0.0440	9.667
293.150	3.0361	0.6831	0.0561	8.214
293.150	4.0352	0.9187	0.0732	7.971
293.150	5.0384	1.2287	0.0817	6.648
293.150	6.0370	1.3239	0.0659	4.974
293.150	11.0546	1.8945	0.0986	5.204
293.150	21.0616	2.3613	0.1243	5.263
293.150	31.0552	3.0605	0.1449	4.735
293.150	41.0640	4.3528	0.2049	4.707
293.150	51.1230	7.5230	0.3360	4.467

Table S3 Experimental results for Raman adsorption measurements of pure carbon dioxide on IG3. Recorded temperature, pressure, net adsorption and estimated uncertainties are listed in the table.

T / K	p / bar	q_{net} / mol·kg^{-1}	$U_C(q_{net})$ / mol·kg^{-1}	$U_C(q_{net}) \cdot (q_{net})^{-1}$ / %
293.150	2.0351	0.4953	0.0867	17.499
293.150	3.0361	0.6277	0.1058	16.847
293.150	4.0585	0.7618	0.1245	16.346
293.150	5.0343	0.9149	0.1491	16.300
293.150	6.0524	1.0586	0.1717	16.220
293.150	11.0477	1.5734	0.2586	16.439
293.150	21.0386	2.3839	0.3907	16.389
293.150	31.0689	3.3979	0.5587	16.444
293.150	41.0786	4.6722	0.7724	16.533
293.150	51.0787	8.2900	1.3526	16.316

Table S4 Experimental results for Raman adsorption measurements of (0.4986 carbon dioxide + 0.5014 hydrogen) on IG1. Recorded temperature, pressure, net adsorption and estimated uncertainties are listed in the table.

T / K	p / bar	q_{net} / mol·kg^{-1}	$U_C(q_{net})$ / mol·kg^{-1}	$U_C(q_{net}) \cdot (q_{net})^{-1}$ / %
293.114	2.0418	1.0216	0.1415	13.853
293.150	3.0375	1.5725	0.2083	13.245
293.125	4.0341	1.9180	0.2588	13.496
293.150	5.0315	2.1444	0.2951	13.761
293.150	6.0513	2.3702	0.2492	10.513
293.150	11.0428	3.2134	0.3238	10.077
293.150	21.0571	5.2301	0.5156	9.858
293.150	31.0431	6.4316	0.6308	9.807
293.150	41.0288	7.3386	0.7359	10.028
293.150	51.0408	8.2296	0.8266	10.045

Table S5 Experimental results for Raman adsorption measurements of (0.4986 carbon dioxide + 0.5014 hydrogen) on IG2. Recorded temperature, pressure, net adsorption and estimated uncertainties are listed in the table.

T / K	p / bar	q_{net} / mol·kg^{-1}	$U_C(q_{net})$ / mol·kg^{-1}	$U_C(q_{net}) \cdot (q_{net})^{-1}$ / %
293.150	2.0384	0.9295	0.1117	12.015
293.150	3.0382	1.1085	0.0909	8.200
293.150	4.0357	1.4342	0.0985	6.868
293.150	5.0429	1.6066	0.1108	6.897
293.114	6.0401	1.6562	0.1049	6.334
293.150	11.0465	2.4405	0.1261	5.168
293.150	21.0620	3.4612	0.1585	4.580
293.150	31.0486	4.1469	0.1842	4.442
293.150	41.0226	4.9315	0.2139	4.338
293.150	51.0826	5.6584	0.2864	5.062

Table S6 Experimental results for Raman adsorption measurements of (0.4986 carbon dioxide + 0.5014 hydrogen) on IG3. Recorded temperature, pressure, net adsorption and estimated uncertainties are listed in the table.

T / K	p / bar	q_{net} / mol·kg^{-1}	$U_C(q_{net})$ / mol·kg^{-1}	$U_C(q_{net}) \cdot (q_{net})^{-1}$ / %
293.150	2.0341	0.6427	0.1191	18.539
293.150	3.0343	0.9493	0.1880	19.800
293.150	4.0425	1.1263	0.1925	17.090
293.150	5.0289	1.2013	0.2327	19.375
293.150	6.0259	1.3739	0.2314	16.844
293.150	11.0340	2.0125	0.3365	16.722
293.150	21.0728	2.9281	0.4720	16.120
293.150	31.0399	3.7003	0.6025	16.282
293.150	41.0742	4.4106	0.7190	16.301
293.150	51.0981	5.2480	0.8642	16.467

Table S7 Experimental results for gravimetric adsorption measurements of pure carbon dioxide on IG1. Recorded temperature, pressure, net adsorption and estimated uncertainties are listed in the table.

T / K	p / bar	q_{net} / mol·kg^{-1}	$U_C(q_{net})$ / mol·kg^{-1}	$U_C(q_{net}) \cdot (q_{net})^{-1}$ / %
293.224	0.1119	0.0872	0.0150	17.178
293.219	0.1979	0.1461	0.0152	10.375

293.226	0.3181	0.2146	0.0155	7.199
293.227	0.3948	0.2562	0.0157	6.126
293.227	0.5217	0.3207	0.0162	5.037
293.225	0.6049	0.3582	0.0165	4.596
293.218	0.6959	0.3975	0.0168	4.230
293.219	0.7949	0.4410	0.0172	3.911
293.214	0.8898	0.4814	0.0177	3.672
293.223	3.8982	1.3430	0.0313	2.327
293.224	6.9225	1.9370	0.0431	2.224
293.224	9.9098	2.4192	0.0535	2.213
293.226	13.9210	2.9785	0.0667	2.239
293.227	21.9010	3.9805	0.0933	2.345
293.227	25.9280	4.4919	0.1085	2.416
293.228	29.8990	5.0320	0.1257	2.498
293.230	33.9180	5.6362	0.1463	2.595
293.232	37.9530	6.3444	0.1719	2.710
293.240	42.1430	7.2541	0.2071	2.855
293.245	46.1760	8.4214	0.2552	3.030
293.258	50.1670	10.1491	0.3307	3.258
293.274	54.0870	13.6420	0.4869	3.569
293.243	55.0310	15.6195	0.5721	3.663
293.238	56.0030	19.4910	0.7340	3.766
293.225	56.5540	24.1354	0.9244	3.830
293.247	57.1620	26.9411	1.0599	3.934
293.242	57.2610	28.3429	1.1195	3.950
293.240	57.3660	28.6737	1.1389	3.972

Table S8 Experimental results for gravimetric adsorption measurements of pure carbon dioxide on IG2. Recorded temperature, pressure, net adsorption and estimated uncertainties are listed in the table.

T / K	p / bar	q_{net} / mol·kg^{-1}	$U_C(q_{net})$ / mol·kg^{-1}	$U_C(q_{net}) \cdot (q_{net})^{-1}$ / %
293.167	0.1090	0.0953	0.0053	5.506
293.170	0.2170	0.1614	0.0059	3.623
293.170	0.2859	0.1958	0.0063	3.189
293.171	0.4144	0.2523	0.0070	2.775
293.172	0.4969	0.2863	0.0075	2.622
293.172	0.6129	0.3261	0.0081	2.490
293.170	0.6909	0.3522	0.0085	2.426
293.170	0.7979	0.3855	0.0091	2.361
293.170	0.8988	0.4148	0.0096	2.314
293.170	1.9126	0.6489	0.0139	2.135
293.168	3.9263	0.9772	0.0203	2.069
293.164	4.8682	1.1025	0.0288	2.060
293.166	5.9320	1.2289	0.0254	2.055
293.165	6.9269	1.3367	0.0276	2.054
293.165	7.9217	1.4366	0.0298	2.055
293.165	8.9066	1.5298	0.0317	2.057

293.167	9.8938	1.6175	0.0336	2.060
293.185	15.9050	2.0731	0.0442	2.095
293.181	20.9090	2.4088	0.0529	2.143
293.204	30.8660	3.0915	0.0740	2.297
293.197	36.0840	3.5370	0.0902	2.421
293.200	41.0220	4.0926	0.1125	2.578
293.206	45.9880	4.9440	0.1497	2.797
293.220	51.2180	6.8261	0.2360	3.138
293.211	53.8830	9.5397	0.3599	3.391
293.201	55.0380	12.5531	0.4947	3.530
293.195	56.0370	16.1685	0.6662	3.677
293.198	56.6550	16.3718	0.6980	3.786
293.201	57.1610	16.3284	0.7181	3.887

Table S9 Experimental results for gravimetric adsorption measurements of pure carbon dioxide on IG3. Recorded temperature, pressure, net adsorption and estimated uncertainties are listed in the table.

T / K	p / bar	q_{net} / mol·kg^{-1}	$U_C(q_{net})$ / mol·kg^{-1}	$U_C(q_{net}) \cdot (q_{net})^{-1}$ / %
293.212	0.8078	0.2689	0.0272	10.118
293.213	1.8600	0.4810	0.0285	5.931
293.217	2.9111	0.6540	0.0305	4.658
293.221	3.9236	0.7965	0.0326	4.098
293.221	4.8551	0.9165	0.0349	3.805
293.220	5.9196	1.0440	0.0376	3.604
293.220	6.9241	1.1518	0.0403	3.499
293.225	7.9263	1.2641	0.0432	3.420
293.214	8.9156	1.3703	0.0462	3.373
293.205	9.9346	1.4767	0.0494	3.345
293.206	10.9200	1.5780	0.0526	3.332
293.237	20.8620	2.4782	0.0879	3.548
293.244	30.8510	3.4570	0.1334	3.860
293.260	41.2870	4.9242	0.2041	4.144
293.282	51.2340	8.2282	0.3527	4.286
293.271	56.0770	17.5993	0.7232	4.109
293.267	57.2490	43.0007	1.7097	3.976
293.252	57.3680	50.8085	2.0217	3.979

Table S10 Experimental results for gravimetric adsorption measurements of (0.4986 carbon dioxide + 0.5014 hydrogen) on IG1. Recorded temperature, pressure, net adsorption and estimated uncertainties are listed in the table. (Please note: The saturation pressure for the given gas mixture at T = 293.15 K is p_S = 1073,1 bar.)

T / K	p / bar	q_{net} / mol·kg^{-1}	$U_C(q_{net})$ / mol·kg^{-1}	$U_C(q_{net}) \cdot (q_{net})^{-1}$ / %
293.137	0.8991	0.6173	0.0312	5.049
293.157	1.9126	1.0673	0.0358	3.350
293.162	2.8944	1.4203	0.0405	2.852
293.161	3.8943	1.7326	0.0453	2.614
293.156	4.9260	2.0234	0.0501	2.478

293.166	10.9230	3.3469	0.0751	2.245
293.176	20.9190	4.9322	0.1094	2.219
293.179	30.9210	6.1624	0.1395	2.264
293.178	41.1890	7.2411	0.1689	2.333
293.178	51.1530	8.1860	0.1976	2.414
293.177	61.1760	9.0687	0.2274	2.508
293.181	71.2080	9.9103	0.2588	2.611
293.130	61.6790	9.3026	0.2327	2.501
293.123	51.6450	8.3762	0.2019	2.410
293.102	31.1950	6.2586	0.1415	2.261
293.102	11.0610	3.4044	0.0762	2.239
293.131	5.0462	2.0749	0.0510	2.458
293.145	1.0421	0.7084	0.0319	4.509

Table S11 Experimental results for gravimetric adsorption measurements of (0.4986 carbon dioxide + 0.5014 hydrogen) on IG2. Recorded temperature, pressure, net adsorption and estimated uncertainties are listed in the table. (Please note: The saturation pressure for the given gas mixture at T = 293.15 K is p_S = 1073,1 bar.)

T / K	p / bar	q_{net} / mol·kg^{-1}	$U_C(q_{net})$ / mol·kg^{-1}	$U_C(q_{net}) \cdot (q_{net})^{-1}$ / %
293.169	0.1070	0.1057	0.0096	9.076
293.173	0.1860	0.1670	0.0099	5.946
293.175	0.3089	0.2480	0.0106	4.264
293.173	0.3839	0.2934	0.0110	3.758
293.173	0.5169	0.3606	0.0118	3.270
293.174	0.5919	0.3975	0.0123	3.083
293.175	0.6879	0.4413	0.0128	2.908
293.176	0.8126	0.4944	0.0136	2.748
293.176	0.8988	0.5276	0.0141	2.667
293.176	1.8746	0.8393	0.0192	2.287
293.174	2.9160	1.0961	0.0238	2.175
293.175	3.9223	1.3074	0.0278	2.127
293.174	5.8949	1.6559	0.0345	2.085
293.172	6.9177	1.8149	0.0377	2.075
293.167	7.8926	1.9582	0.0405	2.068
293.168	8.9169	2.0970	0.0433	2.063
293.170	9.9396	2.2296	0.0459	2.059
293.171	11.9440	2.4671	0.0507	2.056
293.170	13.9440	2.6853	0.0552	2.055
293.171	15.9480	2.8881	0.0594	2.056
293.171	17.9440	3.0772	0.0634	2.059
293.171	19.9400	3.2547	0.0671	2.062
293.168	23.9560	3.5889	0.0743	2.071
293.169	25.9610	3.7435	0.0778	2.077
293.171	27.9320	3.8914	0.0811	2.083
293.171	29.9390	4.0363	0.0844	2.090
293.171	31.9390	4.1756	0.0876	2.097
293.168	33.9470	4.3121	0.0908	2.105

293.167	36.2760	4.4653	0.0944	2.114
293.168	38.3130	4.5972	0.0976	2.123
293.170	40.2820	4.7211	0.1007	2.132
293.170	42.2600	4.8434	0.1037	2.142
293.169	44.2270	4.9633	0.1068	2.152
293.166	46.2640	5.0857	0.1100	2.163
293.169	48.2550	5.2031	0.1131	2.174
293.167	50.2550	5.3201	0.1163	2.186
293.169	52.2590	5.4346	0.1195	2.198
293.179	56.2000	5.6471	0.1255	2.223
293.168	57.2640	5.7212	0.1276	2.230
293.158	55.4280	5.6345	0.1249	2.217
293.156	53.4100	5.5216	0.1217	2.204
293.159	51.4370	5.4087	0.1186	2.192
293.159	49.4400	5.2925	0.1154	2.180
293.162	47.4520	5.1766	0.1123	2.169
293.163	45.4350	5.0550	0.1091	2.158
293.160	43.4220	4.9334	0.1059	2.147
293.158	41.4170	4.8129	0.1029	2.137
293.162	39.4300	4.6880	0.0998	2.128
293.161	37.4430	4.5652	0.0967	2.119
293.159	35.4600	4.4349	0.0936	2.110
293.161	33.1250	4.2795	0.0899	2.101
293.160	31.1180	4.1417	0.0867	2.093
293.164	29.1160	4.0007	0.0835	2.086
293.161	27.1230	3.8570	0.0802	2.080
293.163	25.0840	3.7010	0.0768	2.074
293.163	23.0930	3.5428	0.0733	2.069
293.164	21.0780	3.3772	0.0697	2.064
293.165	19.0690	3.2040	0.0660	2.060
293.164	17.0790	3.0234	0.0622	2.057
293.167	15.0830	2.8242	0.0580	2.055
293.166	13.0610	2.6188	0.0538	2.054
293.165	11.0480	2.3870	0.0491	2.056
293.163	8.0486	2.0056	0.0414	2.065
293.160	4.0429	1.3651	0.0289	2.117
293.164	1.0398	0.6199	0.0155	2.502

Table S12 Experimental results for gravimetric adsorption measurements of (0.4986 carbon dioxide + 0.5014 hydrogen) on IG3. Recorded temperature, pressure, net adsorption and estimated uncertainties are listed in the table. (Please note: The saturation pressure for the given gas mixture at T = 293.15 K is p_S = 1073,1 bar.)

T / K	p / bar	q_{net} / mol·kg^{-1}	$U_C(q_{net})$ / mol·kg^{-1}	$U_C(q_{net}) \cdot (q_{net})^{-1}$ / %
293.210	0.8068	0.3327	0.0520	15.645
293.209	1.9206	0.6030	0.0530	8.792
293.214	2.8994	0.7908	0.0541	6.841
293.214	3.8883	0.9638	0.0558	5.791

293.215	4.8996	1.1191	0.0576	5.147
293.210	5.9429	1.2817	0.0598	4.663
293.214	6.9166	1.4307	0.0620	4.335
293.210	7.8995	1.5562	0.0642	4.127
293.209	8.9583	1.6895	0.0668	3.953
293.207	9.9391	1.8154	0.0693	3.820
293.233	20.9250	2.8754	0.1001	3.480
293.236	30.9450	3.7150	0.1320	3.552
293.238	41.1680	4.4564	0.1660	3.726
293.238	51.1870	5.1257	0.2009	3.919
293.226	56.1750	5.4788	0.2193	4.002
293.220	57.2500	5.5530	0.2232	4.020
293.217	58.0620	5.6189	0.2264	4.029
293.208	57.4240	5.6041	0.2244	4.005
293.204	56.3980	5.5467	0.2208	3.981
293.182	46.5700	4.8905	0.1855	3.794
293.180	36.4920	4.1757	0.1509	3.614
293.182	26.1320	3.3745	0.1170	3.468
293.180	16.0850	2.4800	0.0863	3.481
293.178	6.0448	1.3358	0.0604	4.520
293.192	1.0397	0.4464	0.0525	11.752
